# Response to: Comment on “Tapered Cuff versus Conventional Cuff for Ventilator-Associated Pneumonia in Ventilated Patients: A Meta-Analysis of Randomized Controlled Trials”

**DOI:** 10.1155/2020/1601785

**Published:** 2020-02-13

**Authors:** Xu An Huang, Wei Min Huang, Yan Ping Du, Liu Xia Li, Fang Fang Wu, Shao Qing Hong, Fang Xuan Tang, Zhang Qiang Ye

**Affiliations:** ^1^Department of Clinical Medicine, Xiamen University Medical College, Xiamen, China; ^2^Department of Hospital Infection-Control, Xiamen Haicang Hospital, Xiamen, China; ^3^Department of Respiratory Medicine, Zhongshan Hospital, Xiamen University, Teaching Hospital of Fujian Medical University, Xiamen, China; ^4^Fujian Medical University, Fuzhou, China

We are very glad to hear from Maertens and Blot [1], and they are as concerned about the same important issues as we are. We also read their excellent paper published in critical care medicine [2].

In the study reported by Mahmoodpoor et al. in 2013 [3], which Maertens and Blot and us both selected, pneumonia was defined according to clinical radiology, laboratory findings, and clinically pulmonary infection score (CPIS), but not based on microbiological confirmation. We know that not all pneumonias in the clinic could be microbiologically confirmed because different doctors perform bronchoscopies in different ways. Sometimes, it is also possible that some doctors perform bronchoalveolar lavage or protective mini-bronchoalveolar lavage at bronchopulmonary segments where the pulmonary patch shadow is caused by heart failure or other noninfectious reasons. Thus, it does not mean that there is no pneumonia when no bacteria are successfully cultured in the lavage. Therefore, pneumonia as diagnosed by microbiological examination only represents a subset of all pneumonia cases. Diagnosis solely based on bacterial cultures of alveolar lavage or protective alveolar lavage often leads to misdiagnosis of pneumonia.

In another study published by Monsel et al. in 2016 [4], in order to be consistent with the other studies we have chosen, we included the pneumonia diagnosed by clinically diagnostic criteria. Monsel's study [4] contains first postoperative pneumonia and second postoperative pneumonia. Maertens and Blot seem to have selected the first postoperative pneumonia episodes confirmed by microbiological examination. However, we only chose the second postoperative pneumonia herein, because we could not add the two postoperative pneumonias together, which would lead to duplication.

In most of the studies included by us, the polyvinylchloride (PVC) cuffs were used, and many other studies suggested that polyurethane- (PU-) tapered cuffs showed no benefits on microaspiration reduction. Thus, only PVC cuffs in Philippart's study reported in 2014 [5] were included in our analysis. However, whether the cuff material might impact tracheal sealing remains to be further investigated.

In Mahmoodpoor's study reported in 2017 [6], the tapped cuffs combined with subglottic secretion drainage (SSD) caused little bias in our study. As we know, Maertens's study also selected the cases in Mahmoodpoor's study published in 2013 [3] and another two studies reported by Saito, in which SSD was also used. These could also cause bias, and there are some other bias that could not be avoided, such as head of bed elevation, cuff pressure, and use of drugs for controlling gastric acid. All of these are known to affect the occurrence of ventilator-associated pneumonia.

Our study only included the published studies but not the unpublished studies.

Finally, we believe that we share with Maertens and Blot [1] the opinion that the tapered-cuff tracheal tube may not be superior to the standard-cuff tracheal tube in reducing ventilator-associated pneumonia (VAP) and intensive care unit (ICU) mortality.
